# Utilization, cost, and specialty distribution of topical ruxolitinib prescribing in Medicare Part D, 2018-2024: A cross-sectional study

**DOI:** 10.1016/j.jdin.2026.08.003

**Published:** 2026-08-23

**Authors:** Joshua A. Kent, Gabriel Santos Malave, Yoni Sacknovitz, Larisa J. Geskin

**Affiliations:** Department of Dermatology, Columbia University Irving Medical Center, New York, New York

**Keywords:** atopic dermatitis, cost analysis, crisaborole, drug utilization, epidemiology, Janus kinase inhibitor, Medicare Part D, prescribing patterns, tacrolimus, topical ruxolitinib, vitiligo

*To the Editor:* Topical ruxolitinib 1.5% cream (Opzelura) is the first U.S. food and drug administration (FDA)-approved topical Janus kinase inhibitor, with indications for atopic dermatitis since September 2021 and nonsegmental vitiligo since July 2022. We used Medicare Part D data to describe national utilization, spending, and prescriber distribution for topical ruxolitinib through 2024, alongside three established nonsteroidal topical antiinflammatories used for atopic dermatitis : crisaborole (Eucrisa, FDA-approved 2016), generic tacrolimus ointment, and generic pimecrolimus cream.

We used the Medicare Part D Prescribers by Provider and Drug Public Use File for 2018-2024.^1^ Records were identified by the brand names Opzelura, Eucrisa, Elidel, and Protopic, and by the generic names pimecrolimus and tacrolimus, with the latter restricted to dermatology and micrographic dermatologic surgery prescribers as a proxy for topical formulation, since the file does not differentiate between routes of administration. This approach is consistent with methods used by Ugwu et al for topical calcineurin inhibitors.^2^ For each drug-year, we calculated the number of unique prescribers, total claims, total days of supply, total drug cost (excluding manufacturer rebates), and cost per day of therapy.

Topical ruxolitinib prescribing expanded rapidly after FDA approval. Accross all specialties, medicare Part D prescribers of topical ruxolitinib grew from 5 in 2021 to 1037 in 2024 (126 to 23,713 claims; $256,075 to $49.6 million); among dermatologist, prescribing grew from 2 to 580 (Table I; Fig 1, *A*). Crisaborole prescribing among dermatologists peaked in 2019 (78 prescribers; 2079 claims) and declined to 40 prescribers and 695 claims by 2024. Among dermatologists, generic tacrolimus claims grew from 32,394 in 2018 to 141,342 in 2024 and generic pimecrolimus claims from 7772 to 15,671, while their cost per day fell from $11.15 to $4.29 and $18.68 to $7.69, respectively.

**Table I:** Annual dermatologist-prescribed medicare Part D utilization of topical ruxolitinib (Opzelura), crisaborole (Eucrisa), and dermatologist-prescribed generic tacrolimus and generic pimecrolimus, 2018-2024

Drug	Year	Prescribers	Claims	Day supply	Total cost, $	CPD, $
Topical ruxolitinib	2021	2	42	1,290	85,732	66.46
Topical ruxolitinib	2022	77	2,033	61,576	4,383,274	71.18
Topical ruxolitinib	2023	342	7,451	229,225	17,049,658	74.38
Topical ruxolitinib	2024	580	13,028	398,695	27,414,694	68.76
Crisaborole	2018	68	1,657	47,533	1,111,887	23.39
Crisaborole	2019	78	2,079	59,361	1,459,542	24.59
Crisaborole	2020	62	1,469	44,660	1,093,167	24.48
Crisaborole	2021	50	1,137	34,082	863,784	25.34
Crisaborole	2022	44	802	24,609	660,345	26.83
Crisaborole	2023	52	852	26,773	764,142	28.54
Crisaborole	2024	40	695	21,419	599,025	27.97
Generic tacrolimus∗	2018	1,432	32,394	879,937	9,814,972	11.15
Generic tacrolimus∗	2019	1,821	43,146	1,184,265	11,210,331	9.47
Generic tacrolimus∗	2020	2,144	50,886	1,447,328	12,401,925	8.57
Generic tacrolimus∗	2021	2,620	66,575	1,915,638	13,945,953	7.28
Generic tacrolimus∗	2022	2,977	78,100	2,286,669	16,253,475	7.11
Generic tacrolimus∗	2023	3,616	101,237	2,994,614	19,251,944	6.43
Generic tacrolimus∗	2024	4,725	141,342	4,227,993	18,150,519	4.29
Generic pimecrolimus	2018	397	7,772	216,129	4,037,204	18.68
Generic pimecrolimus	2019	330	6,653	185,155	3,145,868	16.99
Generic pimecrolimus	2020	519	10,056	284,464	3,947,838	13.88
Generic pimecrolimus	2021	605	11,052	319,491	3,876,273	12.13
Generic pimecrolimus	2022	552	10,789	318,908	3,847,902	12.07
Generic pimecrolimus	2023	641	13,096	391,190	4,158,060	10.63
Generic pimecrolimus	2024	771	15,671	469,970	3,613,012	7.69

Data source: Medicare Part D Prescribers by Provider and Drug Public Use File. Topical ruxolitinib was first FDA-approved in September 2021. CMS suppresses any prescriber-drug-year combination with 10 or fewer claims; reported figures therefore exclude prescribers writing 10 or fewer claims per drug per year.

*CMS*, Centers for Medicare & Medicaid Services; *CPD*, cost per day of therapy.

∗ Generic tacrolimus restricted to dermatology and micrographic dermatologic surgery prescribers as a proxy for topical formulation.

**Fig 1:**
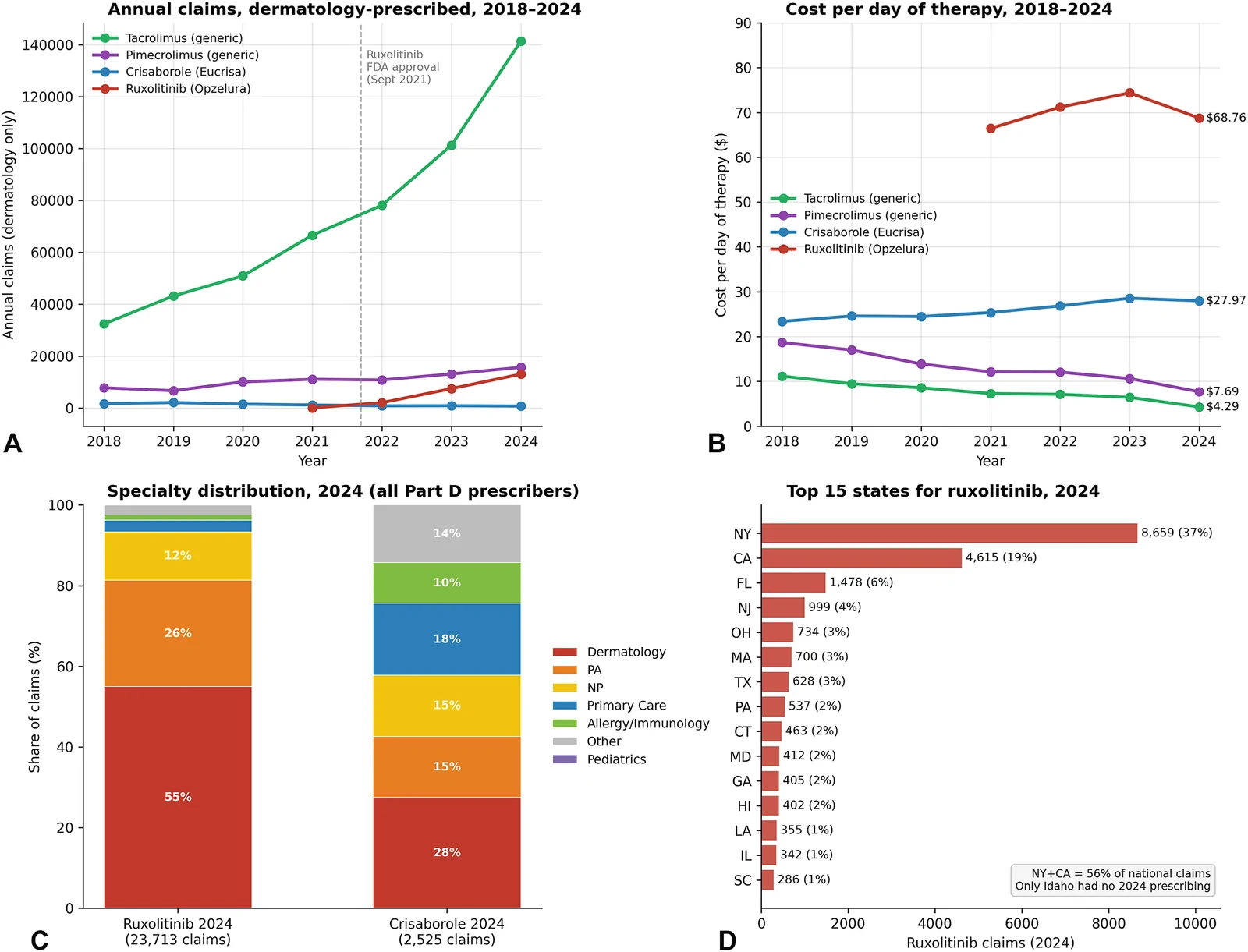
Comparative Medicare Part D prescribing of topical ruxolitinib, crisaborole, and dermatologist-prescribed generic tacrolimus and pimecrolimus, 2018-2024. **A,** Annual claims by drug among dermatology and micrographic dermatologic surgery prescribers; the vertical dashed line indicates FDA approval of topical ruxolitinib in September 2021. **B,** Cost per day of therapy by drug, calculated as total drug cost divided by total day supply; in 2024, topical ruxolitinib cost $68.76 per day, approximately 2.5-fold higher than crisaborole, 8.9-fold higher than generic pimecrolimus, and 16.0-fold higher than generic tacrolimus. **C,** Specialty distribution of 2024 prescribing across all Medicare Part D prescribers. **D,** Top 15 states by topical ruxolitinib claims in 2024; New York and California accounted for 56.0% of national claims, and only Idaho had no Medicare Part D topical ruxolitinib prescribing during 2024.

In 2024, dermatologists wrote 54.9% of topical ruxolitinib claims, physician assistants 26.4%, nurse practitioners 12.0%, primary care physicians 2.9%, and allergy/immunology 1.3% (Fig 1, *C*). For crisaborole, the corresponding shares were 27.5%, 15.0%, 15.2%, 17.7%, and 10.2%.

Among dermatologists in 2024, cost per day of therapy was $68.76 for topical ruxolitinib, $27.97 for crisaborole, $7.69 for generic pimecrolimus, and $4.29 for generic tacrolimus, a 16.0-fold differential between topical ruxolitinib and generic tacrolimus and an 8.9-fold differential versus generic pimecrolimus (Fig 1, *B*). Only 1 of 50 states (Idaho) had no Part D prescribing in 2024, and New York and California together accounted for 56.0% of national claims (Fig 1, *D*).

Prescribing was concentrated among dermatology and dermatology-affiliated advanced practice clinicians, whereas crisaborole is distributed more evenly across specialties. Several factors plausibly contribute, including the drug's recency, its indications, prior authorization requirements, and the Janus kinase class boxed warning; our data cannot distinguish among them. The cost differential is substantial: at 2024 prices, the annual ruxolitinib expenditure would purchase roughly 17,500 patient-years of generic tacrolimus, although these agents are not direct clinical substitutes. The agents also differ in efficacy and tolerability: in pooled phase 3 trials, ruxolitinib achieved Investigator's Global Assessment success in roughly 50% of patients with atopic dermatitis and was well tolerated, whereas tacrolimus 0.1% ointment produced near-clearance (at least 90% improvement on physician global evaluation) in approximately 37% of adults in its pivotal trials, and its tolerability is often limited by application-site burning. These differences may justify a price premium for selected patients.^3^, ^4^, ^5^ Other newer nonsteroidal topicals, such as tapinarof and roflumilast cream, were FDA-approved for atopic dermatitis only in late 2024, after our study period, and warrant dedicated future analysis.

Limitations include Centers for Medicare & Medicaid Services suppression of prescriber-drug-year combinations with 10 or fewer claims, lack of indication or severity data, and the older Medicare population. The public-use file contains no diagnosis codes or patient-level identifiers, so we could not determine the indication for individual prescriptions (atopic dermatitis, vitiligo, or off-label use), severity, or treatment persistence; these questions require linked-claims or chart-based study. Continued surveillance will be important as additional topical Janus kinase inhibitors enter the market and pediatric indications evolve.^3^^,^^5^ In conclusion, topical ruxolitinib achieved rapid Medicare Part D uptake, with a substantial cost premium over existing nonsteroidal topicals and prescribing concentrated within dermatology.

## Conflicts of interest

Dr Geskin has served as an investigator for J&J, Mallinckrodt, Kyowa Kirin, Soligenix, Innate, Incyte, Trillium, Merck, BMS, and Stratpharma; and on the scientific advisory board for SciTech and Citius. The other authors have no conflicts to disclose.
